# Optimized Multilocus Sequence Typing (MLST) Scheme for *Trypanosoma cruzi*

**DOI:** 10.1371/journal.pntd.0003117

**Published:** 2014-08-28

**Authors:** Patricio Diosque, Nicolás Tomasini, Juan José Lauthier, Louisa Alexandra Messenger, María Mercedes Monje Rumi, Paula Gabriela Ragone, Anahí Maitén Alberti-D'Amato, Cecilia Pérez Brandán, Christian Barnabé, Michel Tibayrenc, Michael David Lewis, Martin Stephen Llewellyn, Michael Alexander Miles, Matthew Yeo

**Affiliations:** 1 Unidad de Epidemiología Molecular (UEM), Instituto de Patología Experimental, CONICET- Universidad Nacional de Salta, Salta, Argentina; 2 Faculty of Infectious and Tropical Diseases, Department of Pathogen Molecular Biology, London School of Hygiene and Tropical Medicine, London, United Kingdom; 3 Maladies Infectieuses et Vecteurs Ecologie, Génétique, Evolution et Contrôle, MIVEGEC (IRD 224-CNRS 5290-UM1-UM2), IRD Center, Montpellier, France; Institute of Tropical Medicine, Belgium

## Abstract

*Trypanosoma cruzi*, the aetiological agent of Chagas disease possess extensive genetic diversity. This has led to the development of a plethora of molecular typing methods for the identification of both the known major genetic lineages and for more fine scale characterization of different multilocus genotypes within these major lineages. Whole genome sequencing applied to large sample sizes is not currently viable and multilocus enzyme electrophoresis, the previous gold standard for *T. cruzi* typing, is laborious and time consuming. In the present work, we present an optimized Multilocus Sequence Typing (MLST) scheme, based on the combined analysis of two recently proposed MLST approaches. Here, thirteen concatenated gene fragments were applied to a panel of *T. cruzi* reference strains encompassing all known genetic lineages. Concatenation of 13 fragments allowed assignment of all strains to the predicted Discrete Typing Units (DTUs), or near-clades, with the exception of one strain that was an outlier for TcV, due to apparent loss of heterozygosity in one fragment. Monophyly for all DTUs, along with robust bootstrap support, was restored when this fragment was subsequently excluded from the analysis. All possible combinations of loci were assessed against predefined criteria with the objective of selecting the most appropriate combination of between two and twelve fragments, for an optimized MLST scheme. The optimum combination consisted of 7 loci and discriminated between all reference strains in the panel, with the majority supported by robust bootstrap values. Additionally, a reduced panel of just 4 gene fragments displayed high bootstrap values for DTU assignment and discriminated 21 out of 25 genotypes. We propose that the seven-fragment MLST scheme could be used as a gold standard for *T. cruzi* typing, against which other typing approaches, particularly single locus approaches or systematic PCR assays based on amplicon size, could be compared.

## Author Summary

The single-celled parasite *Trypanosoma cruzi* occurs in mammals and insect vectors in the Americas. When transmitted to humans it causes Chagas disease (American trypanosomiasis) a major public health problem. *T. cruzi* is genetically diverse and currently split into six groups, known as TcI to TcVI. Multilocus sequence typing (MLST) is a method used for studying the population structure and diversity of pathogens and involves sequencing DNA of several different genes and comparing the sequences between isolates. Here, we assess 13 *T. cruzi* genes and select the best combination for diversity studies. Outputs reveal that a combination of 7 genes can be used for both lineage assignment and high resolution studies of genetic diversity, and a reduced combination of four loci for lineage assignment. Application of MLST for assigning field isolates of *T. cruzi* to genetic groups and for detailed investigation of diversity provides a valuable approach to understanding the taxonomy, population structure, genetics, ecology and epidemiology of this important human pathogen.

## Introduction

*Trypanosoma cruzi*, the protozoan causative agent of Chagas disease, is a monophyletic and genetically heterogeneous taxon, with at least six phylogenetic lineages formally recognised as Discrete Typing Units (DTUs), TcI–TcVI [1], or near-clades (clades that are blurred by infrequent inter-lineage genetic recombination, [2]). *T. cruzi* is considered to have a predominantly clonal population structure but with at least some intra-lineage recombination [3], [4], [5], [6]. TcI and TcII are the most genetically distant groups, and the evolutionary origins of TcIII and TcIV remain controversial. Based on sequencing of individual nuclear genes Westenberger et al. [7] suggested that an ancient hybridisation event occurred between TcI and TcII followed by a long period of clonal propagation leading to the extant TcIII and TcIV. Alternatively, de Freitas et al. [8] suggested that TcIII and TcIV have a separate evolutionary ancestry with mitochondrial sequences that are similar to each other but distinct from both TcI and TcII. Recently, Flores-Lopez and Machado [9] proposed that TcIII and TcIV have no hybrid origin. Based on the sequence of 32 genes, they strongly suggested that TcI, TcIII and TcIV are clustered into a major clade that diverged from TcII around 1–2 millions of years ago. Less controversially, it is clear that TcV and TcVI, both overwhelmingly represented in the domestic transmission cycles in the Southern Cone region of South America, are hybrid lineages sharing haplotypes from both TcII and TcIII, with both DTUs retaining the mitochondrial genome of TcIII [8], [10]. Recent phylogenetic studies suggest that the emergence of the hybrid lineages TcV and TcVI may have occurred within the last 60,000 years [11]. Reliable DTU identification and the potential for high resolution investigation of genotypes at the intra DTU level are of great interest for epidemiological, host association, clinical and phylogenetic studies. Historically, a plethora of typing techniques have been applied to *T. cruzi*. Initial pioneering work applied multilocus enzyme electrophoresis (MLEE) techniques [12], [13], [14], [15], [16], [17], [18], [19], [20] revealing the remarkable genetic heterogeneity of this parasite. Subsequently, several PCR-based typing assays have been designed to differentiate the main DTUs [21], [22], [23], [24] and more recently, combinations of PCR-RFLP schemes have been published [25], [26], [27]. Some approaches based on DTU characterisation by direct sequential PCR amplifications from blood and tissue samples are also promising, although various sensitivity and reliability issues need to be resolved [28], [29], [30]. Microsatellite typing (MLMT) has also been applied to population data for fine-scale intra DTU genetic analysis [31], [32], [33].

Multilocus sequence typing (MLST), originally developed for bacterial species typing, has now been applied to a wide range of prokaryotic [34], [35], [36], [37] and increasingly eukaryotic microorganisms [38], [39], [40], [41], [42], [43], [44], [45], [46], [47], [48]. The technique typically involves the sequencing and concatenation of six to ten internal fragments of single copy housekeeping genes per strain [49]. Data are often hosted on interactive open access databases such as MLST.net for use in the wider research community. A major advantage of MLST analysis is that sequence data are unambiguous, minimizing interpretative errors. In this context, the MLST approach represents an excellent candidate to become the gold standard for *T. cruzi* genetic typing with outputs suitable for phylogenetic and epidemiological studies, particularly where large numbers of isolates from varied sources are under study.

Recently, two multilocus sequence typing (MLST) schemes have been developed in parallel for *T. cruzi*, each of them based on different gene targets [50],[51]. Both schemes display a high discriminatory power and are able to clearly differentiate the main *T. cruzi* DTUs. The current work proposes to resolve the optimum combination of loci across the two schemes to produce a reproducible and robust formalised MLST scheme validated across a shared reference panel of isolates for practical use by the wider *T. cruzi* research community.

## Methods

### Parasite strains and DNA isolation

Twenty five cloned reference strains belonging to the six known DTUs were examined (Table 1). These strains have been widely used as reference strains in many previous studies, and are regularly examined in our laboratory by Multilocus Enzyme Electrophoresis (MLEE). Parasite stocks were cultivated at 28°C in liver infusion tryptose (LIT) supplemented with 1% hemin, 10% fetal bovine serum, 100 units/ml of penicillin, and 100 µg/mL of streptomycin or in supplemented RPMI liquid medium.

**Table 1:** Cohort of clonal reference isolates representing the six known *T. cruzi* lineages (DTUs).

Strain	DTU	Origin	Host
**1. X10cl1**	TcI	Belém, Brazil	*Homo sapiens*
**2. Cutia c1**	Tcl	Espiritu Santo, Brazil	*Dasyprocta aguti*
**3. Sp104 cl1**	Tcl	Region IV, Chile	*Triatoma spinolai*
**4. P209 cl93**	Tcl	Sucre, Bolivia	*Homo sapiens*
**5. OPS21 cl11**	Tcl	Cojedes, Venezuela	*Homo sapiens*
**6. 92101601P cl1**	TcI	Georgia, USA	*Didelphis marsupialis*
**7. TU18 cl93**	TcII	Potosí, Bolivia	*Triatoma infestans*
**8. CBB cl3**	TcII	Region IV, Chile	*Homo sapiens*
**9. Mas cl1**	TcII	Federal District, Brazil	*Homo sapiens*
**10. IVV cl4**	TcII	Region IV, Chile	*Homo sapiens*
**11. Esm cl3**	TcII	Sào Felipe, Brazil	*Homo sapiens*
**12. M5631 cl5**	TcIII	Selva Terra, Brazil	*Dasypus novemcinctus*
**13. M6241 cl6**	TcIII	Belem, Brazil	*Homo sapiens*
**14. CM17**	TcIII	Meta, Colombia	*Dasypus sp.*
**15. X109/2**	TcIII	Makthlawaiya, Paraguay	*Canis familiaris*
**16. 92122102R**	TcIV	Georgia, USA	*Procyon lotor*
**17. CanIII cl1**	TcIV	Belém, Brazil	*Homo sapiens*
**18. Dog Theis**	TcIV	USA	*Canis familiaris*
**19. Mn cl2**	TcV	Region IV, Chile	*Homo Sapiens*
**20. Bug 2148 cl1**	TcV	Rio Grande do sul, Brazil	*Triatoma infestans*
**21. SO3 cl5**	TcV	Potosi, Bolivia	*Triatoma infestans*
**22. SC43 cl1**	TcV	Santa-Cruz, Bolivia	*Triatoma infestans*
**23. CL Brener**	TcVI	Rio Grande do Sul, Brazil	*Triatoma infestans*
**24. P63 cl1**	TcVI	Makthlawaiya, Paraguay	*Triatoma infestans*
**25. Tula cl2**	TcVI	Talahuen, Chile	*Homo sapiens*

### MLST loci

Initially a total of 19 gene fragments were considered, 10 housekeeping genes previously described by Lauthier et al. [50] [Glutathione peroxidase (*GPX*), 3-Hidroxi-3-metilglutaril-CoA reductase (*HMCOAR*), Piruvate dehydrogenase component E1 subunit alfa (*PDH*), Small GTP-binding protein Rab7 (*GTP*), Serine/treonine-protein phosphatase PP1 (*STPP2*), Rho-like GTP binding protein (*RHO1*), Glucose-6-phosphate isomerase (*GPI*), Superoxide dismutase A (*SODA*), Superoxide dismutase B (*SODB*) and Leucine aminopeptidase (*LAP*)]; and 9 gene fragments from Yeo et al. [51] [ascorbate-dependent haemoperoxidase (*TcAPX*), dihydrofolate reductase-thymidylate synthase (*DHFR-TS*), glutathione-dependent peroxidase II (*TcGPXII*), mitochondrial peroxidase (*TcMPX*), trypanothione reductase (*TR*), RNA-binding protein-19 (*RB19*), metacyclin-II (*Met-II*), metacyclin-III (*Met-III*) and *LYT1*]. However, 6 of them were discarded based on initial findings [50], [51]. Although some of the excluded targets were informative, they were not amenable for routine use. More specifically, *LYT1* was discarded due to unreliable PCR amplification and sequencing despite multiple attempts at optimization; *TR*, *DHFR-TS* and *TcAPX* were also deemed unsuitable as internal sequencing primers were required; finally, *Met-III* and *TcGPXII* were also excluded because generated non-specific PCR products with some isolates.

The final 13 gene fragments assessed included 3 fragments described by Yeo et al. [51] and the 10 housekeeping genes previously described by Lauthier et al. [50]. These were: *TcMPX*, *RB19*, *Met-II*, *SODA*, *SODB*, *LAP*, *GPI*, *GPX*, *PDH*, *HMCOAR*, *RHO1*, *GTP* and *STPP2*. For the 13 loci under study, searches in the CL-Brener and Sylvio X10 genomes (http://tritrypdb.org/tritrypdb/), using the primer sequences as well as the fragment sequences as query, displayed single matches in all cases. Chromosome location, primer sequences and amplicon size for each target are shown in Table 2. Nucleotide sequences for all the analysed MLST targets are available from GenBank under the following accession numbers: JN129501-JN129502, JN129511-JN129518, JN129523-JN129524, JN129534-JN129535, JN129544-JN129551, JN129556-JN129557, JN129567-JN129568, JN129577-JN129584, JN129589-JN129590, JN129600-JN129601, JN129610-JN129617, JN129622-JN129623, JN129633-JN129634, JN129643- JN129650, JN129655-JN129656, JN129666-JN129667, JN129676-JN129683, JN129688-JN129689, JN129699-JN129700, JN129709-JN129716, JN129721-JN129722, JN129732-JN129733, JN129742-JN129749, JN129754-JN129755, JN129765-JN129766, JN129775-JN129782, JN129787-JN129788, JN129798-JN129799, JN129808-JN129815, JN129820-JN129821, KF889442-KF889646. Additionaly, we used *T. cruzi marinkellei* as outgroup. Sequence data of the selected targets for *T. cruzi marinkellei* were obtained from TriTrypDB (http://tritrypdb.org), under the following accession Ids: TcMARK_CONTIG_2686, TcMARK_CONTIG_670, TcMARK_CONTIG_1404, Tc_MARK_2068, Tc_MARK_3409, Tc_MARK_5695, Tc_MARK_9874, Tc_MARK_515, Tc_MARK_4984, Tc_MARK_5926, Tc_MARK_8923, TcMARK_CONTIG_1818 and Tc_MARK_2666.

**Table 2:** Details of gene targets.

Gene	Gene ID^a^	Chromosome Number	Primer Sequence (5′-3′)	Amplicon size (bp)^b^	Sequence start 5′^c^	Fragment Length (bp)^d^
***GPI*** * †	Tc00.1047053506529.508	6	CGCCATGTTGTGAATATTGG (20)	405	21	365
			GGCGGACCACAATGAGTATC (20)			
***HMCOAR*** * †	TC00.1047053506831.40	32	AGGAGGCTTTTGAGTCCACA (20)	554	21	514
			TCCAACAACACCAACCTCAA (20)			
***RHO1*** * †	Tc00.1047053506649.40	8	AGTTGCTGCTTCCCATCAAT (20)	455	21	415
			CTGCACAGTGTATGCCTGCT (20)			
***Tc MPX*** * †	Tc00.1047053509499.14	22	ATGTTTCGTCGTATGGCC (18)	678	109	505
			TGCGTTTTTCTCAAAATATTC (21)			
***LAP*** *	Tc00.1047053508799.240	27	TGTACATGTTGCTTGGCTGAG (21)	444	22	402
			GCTGAGGTGATTAGCGACAAA (21)			
***SODB*** *	Tc00.1047053507039.10	35	GCCCCATCTTCAACCTT (17)	313	18	266
			TAGTACGCATGCTCCCATA (19)			
***RB19*** *	Tc00.1047053507515.60	29	GCCTACACCGAGGAGTACCA (20)	408	49	340
			TTCTCCAATCCCCAGACTTG (20)			
***GPX***	Tc00.1047053511543.60	35	CGTGGCACTCTCCAATTACA (20)	360	21	321
			AATTTAACCAGCGGGATGC (19)			
***PDH***	Tc00.1047053507831.70	40	GGGGCAAGTGTTTGAAGCTA (20)	491	21	451
			AGAGCTCGCTTCGAGGTGTA (20)			
***GTP***	Tc00.1047053503689.10	12	TGTGACGGGACATTTTACGA (20)	561	21	521
			CCCCTCGATCTCACGATTTA (20)			
***SODA***	Tc00.1047053509775.40	21	CCACAAGGCGTATGTGGAC (19)	300	20	263
			ACGCACAGCCACGTCCAA (18)			
***STPP2***	Tc00.1047053507673.10	34	CCGTGAAGCTTTTCAAGGAG (20)	409	21	369
			GCCCCACTGTTCGTAAACTC (20)			
***Met-II***	TC00.1047053510889.280	6	TCATCTGCACCGATGAGTTC (20)	700	51	389
			CTCCATAGCGTTGACGAACA (20)			

*Gene fragments included in the 7 loci MLST scheme;

† Gene fragments included in the reduced 4 loci MLST scheme;

a Gene ID: GenBank access number for the complete gene in the CL-Brener strain;

b Amplicon size refers to the sequence size of the gene fragment including the primers regions;

c 5′ starting position: indicates the position where the analyzed sequence starts, counting from the first base of the amplicon;

d Fragment Length refers to the sequence length used for the analyses (the analyzed fragments do not include the primer regions).

### Molecular methods

PCRs were performed in 50 µl reaction volumes containing 100 ng of DNA, 0.2 µM of each primer, 1 U of goTaq DNA polymerase (Promega), 10 µl of buffer (supplied with the GoTaq polymerase) and a 50 µM concentration of each deoxynucleoside triphosphate (Promega). Amplification conditions for all targets were: 5 min at 94°C followed by 35 cycles of 94°C for 1 min; 55°C 1 min, and 72°C for 1 min, with a final extension at 72°C for 5 min. Amplified fragments were purified (QIAquick, Qiagen) and sequenced in both directions (ABI PRISM 310 Genetic Analyzer or ABI PRISM 377 DNA Sequencers, Applied Biosystems) using standard protocols. Primers used for sequencing were identical to those used in PCR amplifications. In order to assess reproducibility, each PCR amplification was performed multiple times and associated sequencing was repeated at least twice.

### Data analysis

MLST data were analysed with MLSTest software (http://ipe.unsa.edu.ar/software) [52] with the objective of identifying the most resolutive and minimum number of targets for unequivocal DTU assignment and potential fine scale characterisation. MLSTest contains a suite of MLST data specific analytical tools. Briefly, single nucleotide polymorphisms (SNPs) were identified in all loci in MLSTest alignment viewer. Typing efficiency (TE) was calculated using the same software. TE for a determined locus is calculated as the number of identified genotypes divided by the number of polymorphic sites in this locus. Additionally, discriminatory power, defined as the probability that two strains are distinguished when chosen at random from a population of unrelated strains [53], was determined for each target (Table 3).

**Table 3:** *T. cruzi* MLST targets.

Gene fragment	No. of genotypes	No. of polymorphic sites	Typing efficiency^1^	DP
***GPI*** * †	9	18	0.500	0.889
***HMCOAR*** * †	15	20	0.750	0.954
***RHO1*** * †	13	23	0.565	0.914
***Tc MPX*** * †	11	12	0.917	0.905
***LAP*** *	13	16	0.812	0.942
***SODB*** *	12	9	1.333	0.914
***RB19*** *	21	26	0.808	0.985
***GPX***	12	16	0.750	0.908
***PDH***	11	15	0.733	0.920
***GTP***	10	18	0.556	0.905
***SODA***	10	10	1.000	0.880
***STPP2***	5	8	0.625	0.585
***Met-II***	19	40	0.475	0.978

DP: Discriminatory Power according to [53],

1 Number of genotypes per polymorphic site,

*Included in the seven loci scheme,

† Included in the four loci scheme.

Sequence data were concatenated and Neighbour Joining phylogenetic trees were generated by using uncorrected p-distances. Heterozygous sites were handled in the analyses using two different methods. First, a SNP duplication method described by Yeo et al. and Tavanti et al. [51], [54] was implemented. Briefly, the SNP duplication method involves the elimination of monomorphic sites and duplication of polymorphisms in order to “resolve” the heterozygous sites. As an example, a homozygous variable locus scored as C (cytosine) will be modified by CC; while a heterozygous locus, for example Y (C or T, in accordance with IUPAC nomenclature), will be scored as CT. Alternatively, heterozygous SNPs were considered as average states. In more detail, the genetic distance between T and Y (heterozygosity composed of T and C) is considered as the mean distance between the T and the possible resolutions of Y (distance T-T = 0 and distance T-C = 1, average distance = 0.5, see [53] and MLSTest 1.0 manual at http://www.ipe.unsa.edu.ar/software for further details). Statistical support was evaluated by 1000 bootstrap replications. Overall phylogenetic incongruence among loci (by comparison with the concatenated topology) was assessed by the Incongruence Length Difference Test using the BIO-Neighbour Joining method (BIONJ-ILD, [55]) and evaluated by a permutation test with 1,000 replications. Briefly, the ILD evaluates whether the observed incongruence among fragments is higher than that expected by random unstructured homoplasy across the different fragments. A statistical significant ILD p value indicates that many sites, in at least one fragment, support a phylogeny that is contradicted by other fragments. In order to localize significant incongruent branches in concatenated data we used the Neighbour Joining based Localized Incongruence Length Difference (NJ-LILD) test available in MLSTest. NJ-LILD is a variant of the ILD test that allows localizing incongruence at branch level.

All combinations from 2 to 12 fragments were analysed using the scheme optimisation algorithm in MLSTest which identifies the combination of loci producing the maximum number of diploid sequence types (DSTs). Three main sequential criteria were applied to select the optimum combination of loci: firstly, monophyly of DTUs and lineage assignment; secondly, robust bootstrap values for the six major DTUs (1000 replications); and thirdly detection of genetic diversity at the intra-DTU level.

## Results

### PCR amplification and sequencing

All 13 gene fragments were successfully amplified using identical PCR reaction conditions (see methods) which generated discrete PCR fragments. PCR amplifications of the 13 targets were applied to an extended panel of 90 isolates obtaining more than 98% of positive PCR and amplifications produced strong amplicons and an absence of non-specific products (data not shown). We obtained amplicons of the expected length for all the assayed targets and for all the examined strains. Amplification for various DNA template concentrations was assayed via serial dilution. No difference in PCR amplifications were obtained when DNA concentrations from 20 to 100 ng were used. A total of 5,121 bp of sequence data were analysed for each strain (Table 2). There were no gaps in sequences. The number of polymorphic sites (Table 3) for each of the different fragments varied from 8 (*STPP2*) to 40 (*Met-II*). *STTP2* showed the lowest discriminatory power (describing just 5 different genotypes in the dataset). *Rb19* was the fragment with the highest discriminatory power identifying 21 distinct genotypes in the dataset.

### Optimized scheme for MLST

**Figure 1:**
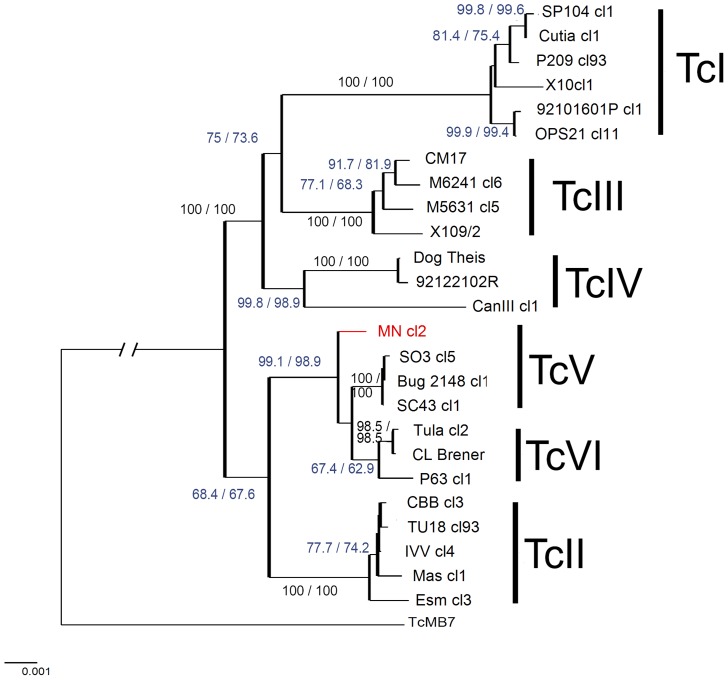
Neighbor Joining tree based on the concatenation of 13 MLST fragments. Different DTUs are represented by vertical bars. Branch values represent bootstrap values (1000 replications), different bootstrap values indicate the method of handling heterozygous sites: SNP duplication method (first value) and average states (second values). Branch supports highlighted in blue shows branches where support for SNP duplication method was higher than the average states method. The outlier TcV is highlighted in red. Scale bar at the bottom left represents uncorrected p-distances.

**Figure 2:**
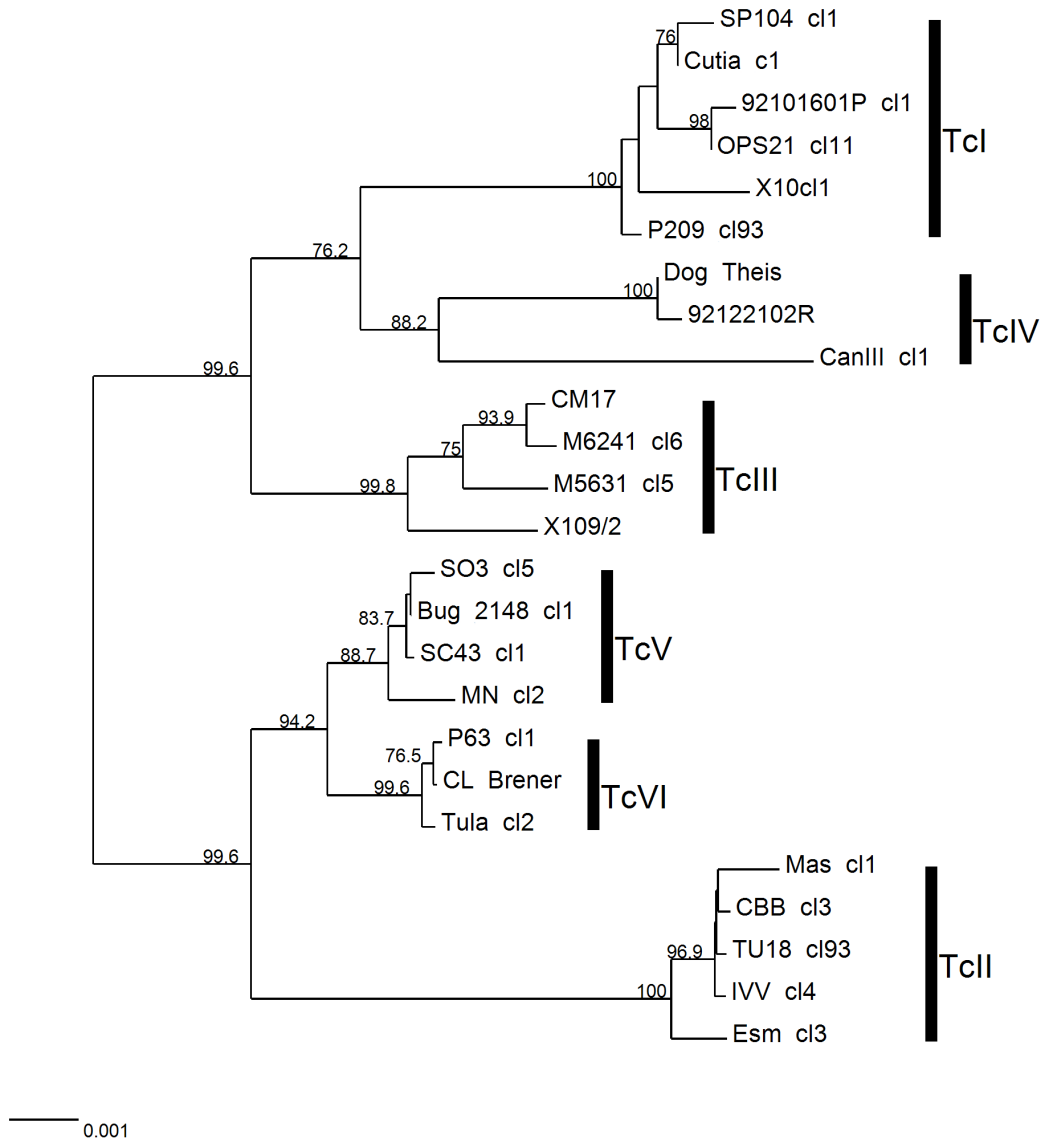
Neighbor Joining tree based on the concatenation of 7 selected MLST fragments: *Rb19*, *TcMPX*, *HMCOAR*, *RHO1*, *GPI*, *SODB* and *LAP*. Different DTUs are represented by vertical bars. Branch values represent bootstrap values (1000 replications). Heterozygous sites were considered as average states (see methods). Scale bar at the bottom left represents uncorrected p-distances.

Initially, Neighbor Joining trees were generated from concatenated sequences across the 13 prescreened loci which identified four monophyletic DTUs with robust bootstrap support (TcI, TcII, TcIII, TcIV, bootstrap >98%). TcVI was also monophyletic but with a relatively low support (Figure 1). Additionally, TcV was paraphyletic with Mncl2 as an outlier. The concatenated 13 fragments differentiated all 25 reference strains in terms of DSTs. We observed that bootstrap values were slightly different between the two methods (SNP duplication and average states) as they manage heterozygous sites differently. Values were higher for the SNP duplication method in most branches (Figure 1, branch values highlighted in blue) as a consequence of base duplication, which modifies the alignment and increases the informative sites used for bootstrapping. To avoid the potential for methodologically elevated bootstraps, the average states method was implemented for further analyses. From the selected 13 loci, all possible combinations of 2 to 12 loci were analysed (8,177 combinations) by implementing the MLSTest scheme optimisation algorithm. One combination of 7 loci was the best according to the proposed criteria. This combination consisted of *Rb19*, *TcMPX*, *HMCOAR*, *RHO1*, *GPI*, *SODB* and *LAP* discriminating all 25 strains as DSTs, and importantly categorising all separate DTUs as a monophyletic group. DTUs were also well-supported by associated bootstraps values (TcI,100; TcII, 100; TcIII, 99.8; TcIV, 88.2; TcV, 88.7; TcVI, 99.6) as illustrated in Figure 2. Combinations with higher number of loci (from 8 to 12) did not significantly increased bootstrap values of TcIV and TcV.

We assessed whether the outlier for TcV (Mn cl2) and the low bootstrap observed for TcVI (applied to all 13 fragments) was due to incongruence among fragments. The thirteen fragment dataset was significantly incongruent (BIONJ-ILD p-value<0.001) for at least one partition which was corroborated using NJ-LILD with a permutation test and 500 replications. Significant incongruence (p-value<0.05 after Bonferroni correction) was detected in the TcV and TcVI nodes. Incongruence was likely due to strains within DTUs TcV and TcVI demonstrating apparent loss of heterozygosis (LOH) in the *Met-II* fragment. Excluding *Met-II*, the p-value for ILD was not significant (BIONJ-ILD p-value = 0.33), and the bootstrap values for TcV and TcVI exceeded 85%, furthermore tree topology was congruent with expected DTU assignment.

### Reduced scheme for DTU assignment

**Figure 3:**
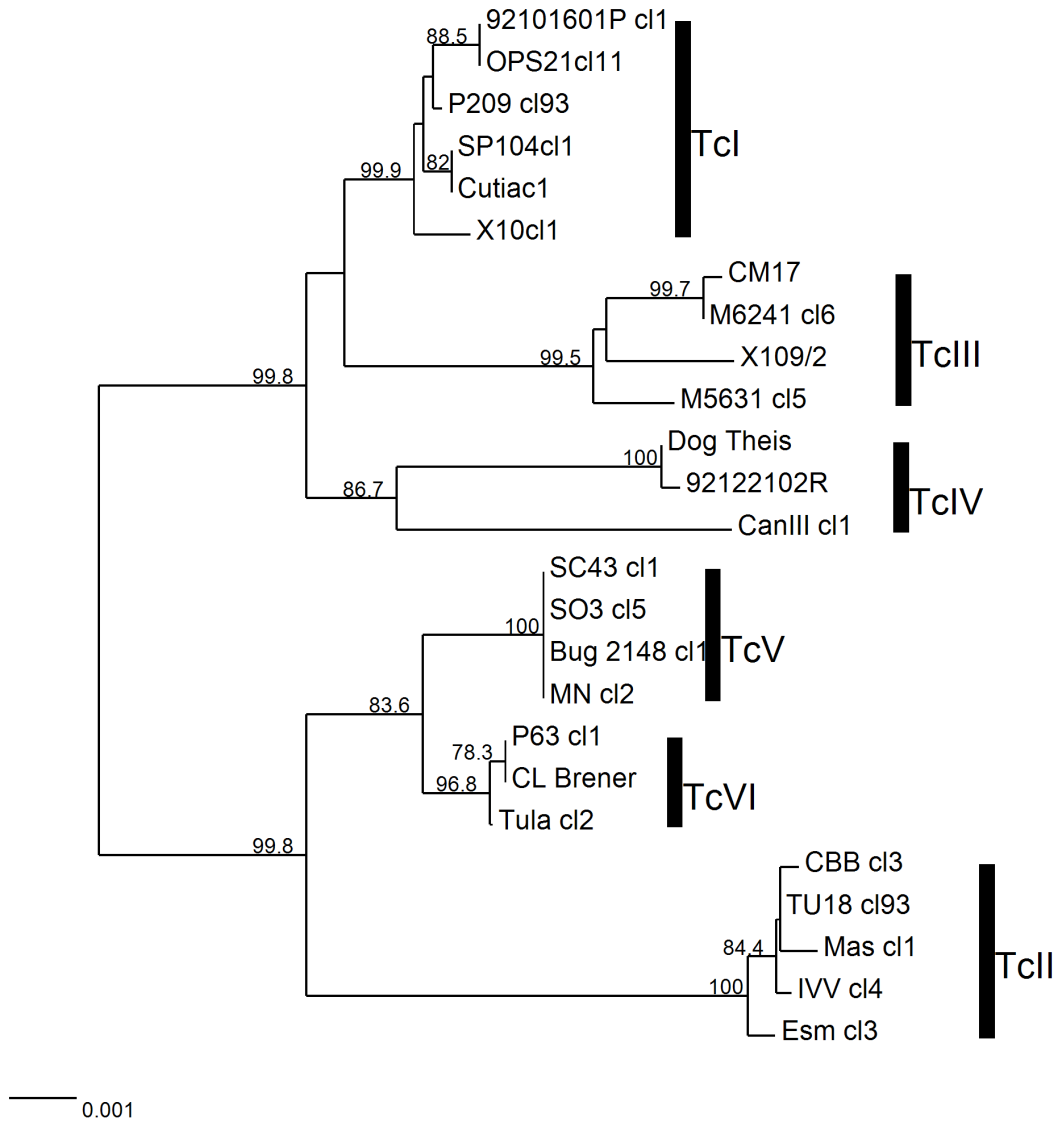
Neighbor Joining tree based on the concatenation of 4 selected MLST fragments (*TcMPX*, *HMCOAR*, *RHO1*, *GPI*) for DTU assignment. Different DTUs are represented by vertical bars. Branch values represent bootstrap values (1000 replications). Heterozygous sites were handled using the average states method. Scale bar at the bottom left represents uncorrected p-distances.

Attempts were made to reduce the number of fragments required for DTU assignment while maintaining DST identification. All combinations of 3 and 4 fragments (1,001 combinations) from the panel of 13 fragments were analysed as described above. A reduced MLST panel incorporating *TcMPX*, *HMCOAR*, *RHO1* and *GPI* (four loci) produced the highest bootstrap values for DTU assignment across the DTUs, TcI (99.9), TcII (100), TcIII (99.5), TcIV (86.7), TcV (100) and TcVI (96.8) (Figure 3), and discriminated 19 of 25 DSTs. Other combinations showed higher discriminatory power but presented with lower bootstrap values (data not shown). The *TcMPX* locus exhibits an apparent loss of heterozygosity (LOH) in the hybrid DTU TcV, retaining the TcII like allele but not the TcIII allele. Therefore DTU assignment using *TcMPX* alone would not assign a TcV isolate correctly. However concatenation of *Tc*MPX with *HMCOAR*, *RHO1* and *GPI* allow distinguishing TcV from TcII.

### Inter and intra DTU phylogenies

Topologies obtained for the 7 and 4 loci combinations (Figures 2 and 3, respectively) were similar to the 13 loci scheme, showing consistently the two major groups (TcI-TcIII-TcIV and TcII-TcV-TcVI) supported by high bootstrap values, even when trees were rooted using TcMB7 (Figure 1). The primary difference between the 13 target concatenated phylogenies and the trees obtained for the 7 and 4 targets was that for the 13 concatenated targets TcV was paraphyletic, showing the Mncl2 strain as an outlier. Regarding inter-DTU relationships, the analysis of the concatenated 13 fragments divided DTUs into two major clusters: one composed by TcI, TcIII and TcIV, with a bootstrap value of 100%; while the remaining group containing TcII, TcV and TcVI was supported by lower bootstrap values (<70%), possibly due to presence of the two hybrid DTUs (TcV and TcVI) (Figure 1). Within clusters, internal topologies were supported with relatively high but variable bootstrap values with 4, 7 and 13 loci combinations and generally consistent intralineage topologies (Figures 1, Figure 2, Figure 3), although the panel of 25 reference strains would need to be expanded further for assessment of fine scale intralineage associations.

## Discussion

Thirteen gene fragments were assessed in an optimised MLST scheme which is a combination of targets from two recently separately proposed schemes [50], [51]. Here we evaluated the optimal combination of loci based on three main sequential criteria: first, assignment to the expected DTU; second, to attain robust bootstrap values for the six major DTUs, and third to detect intra-DTU diversity. For the first time we propose an optimised MLST scheme, validated against a panel representing all known lineages, for characterisation of *T. cruzi* isolates. However, it should be emphasized that this MLST scheme is proposed as a typing method for *T. cruzi* isolates but not as a typing method to be used directly on biological samples as blood, tissues or Triatomine feces, for which more sensitive and simpler methods are needed. Moreover, we have performed assays with the purpose of determining the limit of detection of each gene fragment on blood and triatomines feces (data not shown) and we found that none of these targets are suitable for detecting *T. cruzi* in the normal concentration found in natural biological samples.

As a result of our data analyses, we obtained one combination of 7 loci and one combination of only 4 targets which most closely adhered to the selection criteria described above. It is worth noting that the three used criteria for selecting optimum combination of targets are sequential; it means that there is a hierarchical order of these criteria. In first place, we look for obtaining monophyly for the six DTUs and accurate lineage assignment of each examined strain. In a second place, we look for obtaining robust bootstrap values for each of the six major DTUs. Finally, we expect detecting genetic diversity at the intra-DTU level. In this context, due to the hierarchical order of the criteria of selection of loci, the selected combinations will optimise the number of DSTs but subordinated to the two previous criteria. Theoretically, using these criteria, we could obtain a combination of loci that does not give the maximum number of DST for a determined DTU, because our algorithm previously prioritized obtaining monophyly and strong bootstrap values for the six DTUs. This was the case for the selected 4-loci scheme (which differentiated 19 from 25 strains). In spite of this, the selected 7-loci combination that we propose, allow us to differentiate the 25 examined strains, i.e. the maximum possible number of DSTs. The results illustrate that MLST is a highly discriminatory strain-typing technique. From these data we suggest that the 7 locus scheme provides scope for both lineage assignment and diversity studies, generating robust bootstrap values for distance based phylogenies and that a reduced panel of only four targets is sufficient for assignment to DTU level. For population genetics scale analyses and detailed epidemiological studies a comprehensive larger panel of *T. cruzi* isolates should be assessed by sequencing the proposed targets.

The phylogenetic associations among DTUs TcI, TcII, TcIII and Tc IV are debatable. Split affinities and incongruence have been observed in nuclear phylogenies [7], [8], [51], [56]. One interpretation of phylogenetic incongruence is genetic recombination, although due to the highly plastic nature of the *T. cruzi* genome other causes are also possible. Mutation rates and gene conversion may create distinct levels of sequence diversity [57]. Here, concatenated phylogenies showed a partition into two main clusters for all gene combinations tested, the first consisting of TcI, TcIII and TcIV (bootstrap value = 100%); and the second composed of TcII, TcV and TcVI (bootstrap value <70%). The presence of the two known hybrid lineages (TcV and TcVI) generated artifactual phylogenetic structuring and excluding these representatives revealed clustering of DTUs TcI, TcIII and TcIV, indicating that TcI has a closer affinity to TcIII than to TcIV. TcII is the most genetically distant group which is in agreement with previous findings [9], [10], [51]. In addition, it would be interesting to analyze in the future the new lineage described as TcBat [58] using the MLST scheme proposed here, since it could shed light on the phylogenetical position of this interesting lineage.

LOH observed in *Met-II* and *TcMPX* gene fragments affecting the hybrid lineages TcV and TcVI has potentially significant consequences for MLST and lineage assignment [51]. Isolates affected retain the TcII like allele and would be misassigned in single locus characterisation. For example, hybrid isolates TcV would be assigned to TcII based on *TcMPX* sequencing due to apparent LOH. Despite this LOH the *TcMPX* locus was included in the 4 target scheme to increase bootstrap support in differentiating between TcV from TcVI.

Although MLST has been successfully applied to other diploid organisms including *Candida albicans*, the potential for heterozygous alleles complicates typing schemes. In the present work, two methods to handle heterozygous sites, SNPs duplication and average states algorithms, produced broadly similar results with SNP duplication producing marginally higher bootstraps due to the physical duplication of informative sites. Here we decided to implement the average states methodology to derive genetic distances and phylogenies. Both approaches can be found in the software MLSTest [52] producing results that enable resolution at the DTU level and an associated DP of 1 for the panel tested. A significant advantage of MLST based analysis over sequential PCR based gels is that once generated, sequences can be applied to a range of complementary downstream analyses. For example, the resolution of haplotypes for recombination analysis and investigation of more detailed evolutionary associations can be applied to population sized studies. At present, whole genome sequencing applied to large numbers of isolates is not feasible and microsatellite analysis is often difficult to reproduce precisely across laboratories, unlike MLST which has proven reproducibility both within and between laboratories [59]. However, microsatellites could be more convenient for population genetics studies at a microevolutionary level, due to their high resolution power. A further consideration in the analysis of diploid sequences is differentiating heterozygosity from copy number diversity. Ideally, we should prefer single copy genes for MLST schemes in order to avoid comparisons among paralogous. We performed *in silico* analyses in order to estimate the copy number of the selected targets on the genomic data of CL-Brener (TcVI) and Sylvio X10 (TcI) (http://tritrypdb.org/tritrypdb/). For these analyses, we used as query the primer sequences as well as the complete fragment sequences. These searches displayed just single matches in all cases. Consequently, we propose that all the examined MLST fragments may be considered as single copy genes, at least for typing and clustering.

One of the most important aspects in any MLST scheme is to provide targets that consistently produce PCR amplicons requiring minimal cleanup and are suitable for sequencing. Although in the current protocol, we recommend purifying PCR products with a suitable commercial kit (Quiagen), in most cases, this was not necessary and sequencing was performed directly from the PCR product. The exception was *TcGPXII*, and very occasionally *SODA* produced nonspecific products, neither of which are included in final recommended panels. Although the two previously published MLST [50], [51] schemes showed promise in identifying diversity, some of the gene targets were not amenable for routine use. For example, *LYT1* was discarded due to unreliable amplification and *DHFR-TS* due to the need for internal primers. Therefore further optimisation performed here was necessary for practical use. An important criterion for choosing targets was identifying those that used the same primers for both PCR amplification and sequencing to maintain simplicity and reduce costs.

Taken together, we propose a MLST scheme validated against a panel representing all of the known lineages of *T. cruzi*. We propose that a 7 loci MLST scheme could provide the basis for robust DTU assignment and strain diversity studies of new isolates and a reduced 4 loci scheme for lineage assignment. Importantly, the sequence data generated can be utilised for a wide range of downstream analyses, including the resolution of haplotypes for recombination analysis, population genetics analyses, and other statistical approaches to the phyloepidemiological study of *T. cruzi*.

Finally, we propose that the seven-fragment MLST scheme could be used as a gold standard for *T. cruzi* typing, against which other typing approaches, particularly single locus approaches or systematic PCR assays based on amplicon size, could be compared.

## References

[1] Zingales B, Miles MA, Campbell DA, Tibayrenc M, Macedo AM, et al (2012) The revised *Trypanosoma cruzi* subspecific nomenclature: rationale, epidemiological relevance and research applications. Infect Genet Evol 12: 240–253. 22226704 10.1016/j.meegid.2011.12.009

[2] Tibayrenc M, Ayala FJ (2012) Reproductive clonality of pathogens: A perspective on pathogenic viruses, bacteria, fungi, and parasitic protozoa. Proc Nat Acad Sci USA 109: E3305–E3313. 22949662 10.1073/pnas.1212452109 PMC3511763

[3] Gaunt MW, Yeo M, Frame IA, Stothard JR, Carrasco HJ, et al (2003) Mechanism of genetic exchange in American trypanosomes. Nature 421: 936–939. 12606999 10.1038/nature01438

[4] Lewis MD, Llewellyn MS, Gaunt MW, Yeo M, Carrasco HJ, et al (2009) Flow cytometric analysis and microsatellite genotyping reveal extensive DNA content variation in *Trypanosoma cruzi* populations and expose contrasts between natural and experimental hybrids. Int J Parasitol 39: 1305–1317. 19393242 10.1016/j.ijpara.2009.04.001 PMC2731025

[5] Llewellyn MS, Lewis MD, Acosta N, Yeo M, Carrasco HJ, et al (2009) *Trypanosoma cruzi* IIc: phylogenetic and phylogeographic insights from sequence and microsatellite analysis and potential impact on emergent Chagas disease. PLoS Negl Trop Dis 3: e510. 19721699 10.1371/journal.pntd.0000510 PMC2727949

[6] Messenger LA, Llewellyn MS, Bhattacharyya T, Franzen O, Lewis MD, et al (2012) Multiple mitochondrial introgression events and heteroplasmy in *Trypanosoma cruzi* revealed by maxicircle MLST and next generation sequencing. PLoS Negl Trop Dis 6: e1584. 22506081 10.1371/journal.pntd.0001584 PMC3323513

[7] Westenberger SJ, Barnabe C, Campbell DA, Sturm NR (2005) Two hybridization events define the population structure of *Trypanosoma cruzi* . Genetics 171: 527–543. 15998728 10.1534/genetics.104.038745 PMC1456769

[8] de Freitas JM, Augusto-Pinto L, Pimenta JR, Bastos-Rodrigues L, Goncalves VF, et al (2006) Ancestral genomes, sex, and the population structure of *Trypanosoma cruzi* . PLoS Pathog 2: e24. 16609729 10.1371/journal.ppat.0020024 PMC1434789

[9] Flores-Lopez CA, Machado CA (2011) Analyses of 32 loci clarify phylogenetic relationships among *Trypanosoma cruzi* lineages and support a single hybridization prior to human contact. PLoS Negl Trop Dis 5: e1272. 21829751 10.1371/journal.pntd.0001272 PMC3149036

[10] Machado CA, Ayala FJ (2001) Nucleotide sequences provide evidence of genetic exchange among distantly related lineages of *Trypanosoma cruzi* . Proc Natl Acad Sci U S A 98: 7396–7401. 11416213 10.1073/pnas.121187198 PMC34680

[11] Lewis MD, Llewellyn MS, Yeo M, Acosta N, Gaunt MW, et al (2011) Recent, independent and anthropogenic origins of *Trypanosoma cruzi* hybrids. PLoS Negl Trop Dis 5: e1363. 22022633 10.1371/journal.pntd.0001363 PMC3191134

[12] Barnabe C, Brisse S, Tibayrenc M (2000) Population structure and genetic typing of *Trypanosoma cruzi*, the agent of Chagas disease: a multilocus enzyme electrophoresis approach. Parasitology 120 (Pt 5) 513–526. 10840981 10.1017/s0031182099005661

[13] Miles MA, Cibulskis RE (1986) Zymodeme characterization of *Trypanosoma cruzi* . Parasitol Today 2: 94–97. 15462787 10.1016/0169-4758(86)90037-2

[14] Miles MA, Lanham SM, de Souza AA, Povoa M (1980) Further enzymic characters of *Trypanosoma cruzi* and their evaluation for strain identification. Trans R Soc Trop Med Hyg 74: 221–237. 6992358 10.1016/0035-9203(80)90251-5

[15] Miles MA, Souza A, Povoa M, Shaw JJ, Lainson R, et al (1978) Isozymic heterogeneity of *Trypanosoma cruzi* in the first autochthonous patients with Chagas' disease in Amazonian Brazil. Nature 272: 819–821. 417267 10.1038/272819a0

[16] Miles MA, Toye PJ, Oswald SC, Godfrey DG (1977) The identification by isoenzyme patterns of two distinct strain-groups of *Trypanosoma cruzi*, circulating independently in a rural area of Brazil. Trans R Soc Trop Med Hyg 71: 217–225. 407674 10.1016/0035-9203(77)90012-8

[17] Tibayrenc M, Ayala FJ (1987) [High correlation between isoenzyme classification and kinetoplast DNA variability in *Trypanosoma cruzi*]. C R Acad Sci III 304: 89–92. 3030516

[18] Tibayrenc M, Ayala FJ (1988) Isozyme varibility in *Trypanosoma cruzi*, the agent of Chagas' Disease: Genetical, Taxonomical, and Epidemiological Significance. Evolution 42: 277–292. 28567853 10.1111/j.1558-5646.1988.tb04132.x

[19] Tibayrenc M, Echalar L, Dujardin JP, Poch O, Desjeux P (1984) The microdistribution of isoenzymic strains of *Trypanosoma cruzi* in southern Bolivia; new isoenzyme profiles and further arguments against Mendelian sexuality. Trans R Soc Trop Med Hyg 78: 519–525. 6237474 10.1016/0035-9203(84)90075-0

[20] Tibayrenc M, Le Ray D (1984) General classification of the isoenzymic strains of *Trypanosoma (Schizotrypanum) cruzi* and comparison with *T. (S.) C. marinkellei* and *T. (Herpetosoma) rangeli* . Ann Soc Belg Med Trop 64: 239–248. 6391397

[21] Brisse S, Verhoef J, Tibayrenc M (2001) Characterisation of large and small subunit rRNA and mini-exon genes further supports the distinction of six *Trypanosoma cruzi* lineages. Int J Parasitol 31: 1218–1226. 11513891 10.1016/s0020-7519(01)00238-7

[22] Clark CG, Pung OJ (1994) Host specificity of ribosomal DNA variation in sylvatic *Trypanosoma cruzi* from North America. Mol Biochem Parasitol 66: 175–179. 7984184 10.1016/0166-6851(94)90052-3

[23] Souto RP, Fernandes O, Macedo AM, Campbell DA, Zingales B (1996) DNA markers define two major phylogenetic lineages of *Trypanosoma cruzi* . Mol Biochem Parasitol 83: 141–152. 9027747 10.1016/s0166-6851(96)02755-7

[24] Souto RP, Zingales B (1993) Sensitive detection and strain classification of *Trypanosoma cruzi* by amplification of a ribosomal RNA sequence. Mol Biochem Parasitol 62: 45–52. 8114825 10.1016/0166-6851(93)90176-x

[25] Lewis MD, Ma J, Yeo M, Carrasco HJ, Llewellyn MS, et al (2009) Genotyping of *Trypanosoma cruzi*: systematic selection of assays allowing rapid and accurate discrimination of all known lineages. Am J Trop Med Hyg 81: 1041–1049. 19996435 10.4269/ajtmh.2009.09-0305 PMC2825677

[26] Rozas M, De Doncker S, Adaui V, Coronado X, Barnabe C, et al (2007) Multilocus polymerase chain reaction restriction fragment–length polymorphism genotyping of *Trypanosoma cruzi* (Chagas disease): taxonomic and clinical applications. J Infect Dis 195: 1381–1388. 17397011 10.1086/513440

[27] Burgos JM, Altcheh J, Bisio M, Duffy T, Valadares HM, et al (2007) Direct molecular profiling of minicircle signatures and lineages of *Trypanosoma cruzi* bloodstream populations causing congenital Chagas disease. Int J Parasitol 37: 1319–1327. 17570369 10.1016/j.ijpara.2007.04.015

[28] Burgos JM, Diez M, Vigliano C, Bisio M, Risso M, et al (2010) Molecular identification of *Trypanosoma cruzi* discrete typing units in end-stage chronic Chagas heart disease and reactivation after heart transplantation. Clin Infect Dis 51: 485–495. 20645859 10.1086/655680

[29] Cura CI, Lucero RH, Bisio M, Oshiro E, Formichelli LB, et al (2012) Trypanosoma cruzi discrete typing units in Chagas disease patients from endemic and non-endemic regions of Argentina. Parasitology 139: 516–521. 22309735 10.1017/S0031182011002186

[30] Schijman AG, Vigliano C, Burgos J, Favaloro R, Perrone S, et al (2000) Early diagnosis of recurrence of *Trypanosoma cruzi* infection by polymerase chain reaction after heart transplantation of a chronic Chagas' heart disease patient. J Heart Lung Transplant 19: 1114–1117. 11077230 10.1016/s1053-2498(00)00168-6

[31] Barnabe C, De Meeus T, Noireau F, Bosseno MF, Monje EM, et al (2011) *Trypanosoma cruzi* discrete typing units (DTUs): microsatellite loci and population genetics of DTUs TcV and TcI in Bolivia and Peru. Infect Genet Evol 11: 1752–1760. 21801854 10.1016/j.meegid.2011.07.011

[32] Llewellyn MS, Miles MA, Carrasco HJ, Lewis MD, Yeo M, et al (2009) Genome-scale multilocus microsatellite typing of *Trypanosoma cruzi* discrete typing unit I reveals phylogeographic structure and specific genotypes linked to human infection. PLoS Pathog 5: e1000410. 19412340 10.1371/journal.ppat.1000410 PMC2669174

[33] Macedo AM, Pimenta JR, Aguiar RS, Melo AI, Chiari E, et al (2001) Usefulness of microsatellite typing in population genetic studies of *Trypanosoma cruzi* . Mem Inst Oswaldo Cruz 96: 407–413. 11313654 10.1590/s0074-02762001000300023

[34] Dingle KE, Colles FM, Wareing DR, Ure R, Fox AJ, et al (2001) Multilocus sequence typing system for *Campylobacter jejuni* . J Clin Microbiol 39: 14–23. 11136741 10.1128/JCM.39.1.14-23.2001 PMC87672

[35] Enright MC, Day NP, Davies CE, Peacock SJ, Spratt BG (2000) Multilocus sequence typing for characterization of methicillin-resistant and methicillin-susceptible clones of *Staphylococcus aureus* . J Clin Microbiol 38: 1008–1015. 10698988 10.1128/jcm.38.3.1008-1015.2000 PMC86325

[36] Enright MC, Spratt BG, Kalia A, Cross JH, Bessen DE (2001) Multilocus sequence typing of *Streptococcus pyogenes* and the relationships between emm type and clone. Infect Immun 69: 2416–2427. 11254602 10.1128/IAI.69.4.2416-2427.2001 PMC98174

[37] Nallapareddy SR, Duh RW, Singh KV, Murray BE (2002) Molecular typing of selected *Enterococcus faecalis* isolates: pilot study using multilocus sequence typing and pulsed-field gel electrophoresis. J Clin Microbiol 40: 868–876. 11880407 10.1128/jcm.40.3.868-876.2002 PMC120268

[38] Bougnoux ME, Aanensen DM, Morand S, Theraud M, Spratt BG, et al (2004) Multilocus sequence typing of *Candida albicans*: strategies, data exchange and applications. Infect Genet Evol 4: 243–252. 15450203 10.1016/j.meegid.2004.06.002

[39] Bougnoux ME, Diogo D, Francois N, Sendid B, Veirmeire S, et al (2006) Multilocus sequence typing reveals intrafamilial transmission and microevolutions of *Candida albicans* isolates from the human digestive tract. J Clin Microbiol 44: 1810–1820. 16672411 10.1128/JCM.44.5.1810-1820.2006 PMC1479199

[40] Bougnoux ME, Morand S, d'Enfert C (2002) Usefulness of multilocus sequence typing for characterization of clinical isolates of *Candida albicans* . J Clin Microbiol 40: 1290–1297. 11923347 10.1128/JCM.40.4.1290-1297.2002 PMC140389

[41] Bougnoux ME, Tavanti A, Bouchier C, Gow NA, Magnier A, et al (2003) Collaborative consensus for optimized multilocus sequence typing of *Candida albicans* . J Clin Microbiol 41: 5265–5266. 14605179 10.1128/JCM.41.11.5265-5266.2003 PMC262540

[42] Debourgogne A, Gueidan C, Hennequin C, Contet-Audonneau N, de Hoog S, et al (2010) Development of a new MLST scheme for differentiation of *Fusarium solani* Species Complex (FSSC) isolates. J Microbiol Methods 82: 319–323. 20624428 10.1016/j.mimet.2010.07.008

[43] Mauricio IL, Yeo M, Baghaei M, Doto D, Pratlong F, et al (2006) Towards multilocus sequence typing of the *Leishmania donovani* complex: resolving genotypes and haplotypes for five polymorphic metabolic enzymes (ASAT, GPI, NH1, NH2, PGD). Int J Parasitol 36: 757–769. 16725143 10.1016/j.ijpara.2006.03.006

[44] Morehouse EA, James TY, Ganley AR, Vilgalys R, Berger L, et al (2003) Multilocus sequence typing suggests the chytrid pathogen of amphibians is a recently emerged clone. Mol Ecol 12: 395–403. 12535090 10.1046/j.1365-294x.2003.01732.x

[45] Odds FC (2010) Molecular phylogenetics and epidemiology of *Candida albicans* . Future Microbiol 5: 67–79. 20020830 10.2217/fmb.09.113

[46] Odds FC, Jacobsen MD (2008) Multilocus sequence typing of pathogenic *Candida* species. Eukaryot Cell 7: 1075–1084. 18456859 10.1128/EC.00062-08 PMC2446668

[47] Robles JC, Koreen L, Park S, Perlin DS (2004) Multilocus sequence typing is a reliable alternative method to DNA fingerprinting for discriminating among strains of *Candida albicans* . J Clin Microbiol 42: 2480–2488. 15184424 10.1128/JCM.42.6.2480-2488.2004 PMC427821

[48] Zhang CY, Lu XJ, Du XQ, Jian J, Shu L, et al (2013) Phylogenetic and evolutionary analysis of chinese leishmania isolates based on multilocus sequence typing. PLoS One 8: e63124. 23646184 10.1371/journal.pone.0063124 PMC3639960

[49] Maiden MC (2006) Multilocus sequence typing of bacteria. Annu Rev Microbiol 60: 561–588. 16774461 10.1146/annurev.micro.59.030804.121325

[50] Lauthier JJ, Tomasini N, Barnabe C, Rumi MM, D'Amato AM, et al (2012) Candidate targets for Multilocus Sequence Typing of *Trypanosoma cruzi*: validation using parasite stocks from the Chaco Region and a set of reference strains. Infect Genet Evol 12: 350–358. 22210092 10.1016/j.meegid.2011.12.008

[51] Yeo M, Mauricio IL, Messenger LA, Lewis MD, Llewellyn MS, et al (2011) Multilocus Sequence Typing (MLST) for Lineage Assignment and High Resolution Diversity Studies in *Trypanosoma cruzi* . PLoS Negl Trop Dis 5: e1049. 21713026 10.1371/journal.pntd.0001049 PMC3119646

[52] Tomasini N, Lauthier JJ, Llewellyn M, Diosque P (2013) MLSTest: novel software for multi-locus sequence data analyses in eukaryotic organisms. Infect Genet Evol 20: 188–196. 24025589 10.1016/j.meegid.2013.08.029

[53] Hunter PR (1990) Reproducibility and indices of discriminatory power of microbial typing methods. J Clin Microbiol 28: 1903–1905. 2229371 10.1128/jcm.28.9.1903-1905.1990 PMC268075

[54] Tavanti A, Davidson AD, Johnson EM, Maiden MC, Shaw DJ, et al (2005) Multilocus sequence typing for differentiation of strains of *Candida tropicalis* . J Clin Microbiol 43: 5593–5600. 16272492 10.1128/JCM.43.11.5593-5600.2005 PMC1287820

[55] Zelwer M, Daubin V (2004) Detecting phylogenetic incongruence using BIONJ: an improvement of the ILD test. Mol Phylogenet Evol 33: 687–693. 15522796 10.1016/j.ympev.2004.08.013

[56] Rozas M, De Doncker S, Coronado X, Barnabe C, Tibyarenc M, et al (2008) Evolutionary history of *Trypanosoma cruzi* according to antigen genes. Parasitology 135: 1157–1164. 18700995 10.1017/S0031182008004794

[57] Cerqueira GC, Bartholomeu DC, DaRocha WD, Hou L, Freitas-Silva DM, et al (2008) Sequence diversity and evolution of multigene families in *Trypanosoma cruzi* . Mol Biochem Parasitol 157: 65–72. 18023889 10.1016/j.molbiopara.2007.10.002

[58] Marcili A, Lima L, Cavazzana M, Junqueira AC, Veludo HH, et al (2012) A new genotype of Trypanosoma cruzi associated with bats evidenced by phylogenetic analyses using SSU rDNA, cytochrome b and Histone H2B genes and genotyping based on ITS1 rDNA. Parasitology 136: 641–655. 10.1017/S0031182009005861 19368741

[59] Tavanti A, Davidson AD, Fordyce MJ, Gow NA, Maiden MC, et al (2005) Population structure and properties of *Candida albicans*, as determined by multilocus sequence typing. J Clin Microbiol 43: 5601–5613. 16272493 10.1128/JCM.43.11.5601-5613.2005 PMC1287804

